# Toxic Flatworm Egg Plates Serve as a Possible Source of Tetrodotoxin for Pufferfish

**DOI:** 10.3390/toxins11070402

**Published:** 2019-07-11

**Authors:** Taiki Okabe, Hikaru Oyama, Maho Kashitani, Yuta Ishimaru, Rei Suo, Haruo Sugita, Shiro Itoi

**Affiliations:** Department of Marine Science and Resources, Nihon University, Fujisawa, Kanagawa 252-0880, Japan

**Keywords:** egg plates, flatworm, *Planocera multitentaculata*, pufferfish, tetrodotoxin (TTX)

## Abstract

The pufferfish *Takifugu niphobles* (at present *Takifugu alboplumbeus*) possesses highly concentrated tetrodotoxin (TTX), an extremely potent neurotoxin that provides effective protection from predators, at least at the larval stages. However, the source of the toxin has remained unclear. Recently, DNA from the toxic flatworm *Planocera multitentaculata* was detected in the intestinal contents of juveniles and young of the pufferfish, suggesting that the flatworm contributes to its toxification at various stages of its life. In this study, we describe the behavior of the pufferfish in the intertidal zone that appears to contribute to its toxification before and during its spawning period: pufferfish were found to aggregate and ingest flatworm egg plates by scraping them off the surface of rocks. DNA analysis based on 28S rRNA and cytochrome *c* oxidase subunit I (COI) genes identified the egg plates as those of *P. multitentaculata*. Liquid chromatography with tandem mass spectrometry analysis revealed that the egg plates contain highly concentrated TTX. The feeding behavior of the pufferfish on the flatworm egg plates was also observed in the aquarium. These results suggest that pufferfish feed on the flatworm egg plate, which enables them to acquire toxicity themselves while providing their offspring with the protective shield of TTX.

## 1. Introduction

Tetrodotoxin (TTX), also known as pufferfish toxin, is one of the most potent biological toxins. Its mode of action is by inhibiting neurotransmission by means of blocking voltage-gated sodium channels on excitable membranes of muscle and nerve tissues [1,2]. TTX has been found in organisms from extremely diverse taxa, including amphibians (atelopid frog *Atelopus* spp. [3]; newts [4]), fish (pufferfish *Takifugu* spp. [5]; goby *Yongeichthys criniger* [6]), crustaceans [7], cephalopods [8], gastropods [9], bivalves [10,11], flatworms [12,13,14,15], ribbonworms [16,17] and marine bacteria [18,19].

Among these TTX-bearing organisms, pufferfish of the genus *Takifugu*, in particular, have the reputation of possessing the toxin in abundance [5]. It is believed that the TTX accumulates in the pufferfish body via the food chain, beginning with bacteria as the primary producers [5,20]. This conclusion has been inferred by means of several studies where pufferfish fed non-toxic diets after hatching in artificial culture environments, turn out to be non-toxic, and furthermore, these cultured non-toxic pufferfish can then be toxified by means of oral administration of TTX [5,20,21,22,23]. On the other hand, tracing the toxification via the food webs exclusively to marine bacteria is unlikely to account for the amount of TTX in the pufferfish body, as bacteria have been shown to produce rather minute amounts of toxin [24,25,26,27], although TTX levels produced by bacterial cultures (as done in these studies) under potentially non-optimum conditions are likely to be significantly less than those synthesized by bacteria in the natural environment. Recently, our lab reported that the pufferfish *Takifugu niphobles* (at present *Takifugu alboplumbeus*) feeds on the toxic eggs of another pufferfish *T. pardalis*, suggesting that *T. niphobles* effectively toxifies itself by ingesting concentrated TTX from the eggs of related species [28]. Nevertheless, it is important to further explore the source of TTX in pufferfish to fully understand its natural history, especially in relation to the source(s) of TTX, to explain its large reserves.

In another paper, our lab recently reported that the pufferfish *T. niphobles* (at present *Takifugu alboplumbeus*) effectively toxified itself by feeding on the flatworm *P. multitentaculata* [29], suggesting that one of the major sources of TTX in pufferfish was planocerid flatworms. In addition, the flatworm *P. multitentaculata* spawns toxic egg plates on rock and stone surfaces in the intertidal zone from spring to early summer [30,31]. When we investigated the ecological relationship between them in the intertidal zone to understand further the involvement of *P. multitentaculata* in the toxification of the pufferfish, we also noticed another interesting behavior of the pufferfish at the spawning sites of the flatworm. In this study, we describe this behavior of the pufferfish as a possible additional strategy for its toxification.

## 2. Results and Discussion

**Figure 1:**
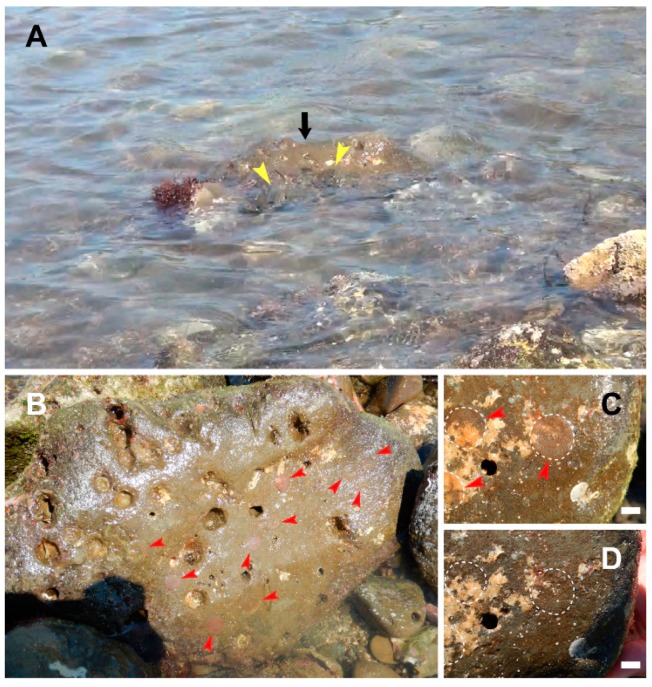
The pufferfish *Takifugu niphobles* (at present *Takifugu alboplumbeus*) feeding on the toxic flatworm *Planocera multitentaculata* egg plates in the wild. Panel **A**, Multiple pufferfish (yellow arrowheads) feeding on the egg plates on rocks. Panel **B**, Exposed rock showing multiple flatworm egg plates (red arrowheads) deposited on the surface. Panels **C** and **D**, Rock surface showing the intact egg plates before pufferfish feeding, and bare rock soon after feeding, respectively. Scale bars, 10 mm. The video of the feeding behavior is provided as supplementary data (Video S1).

**Figure 2:**
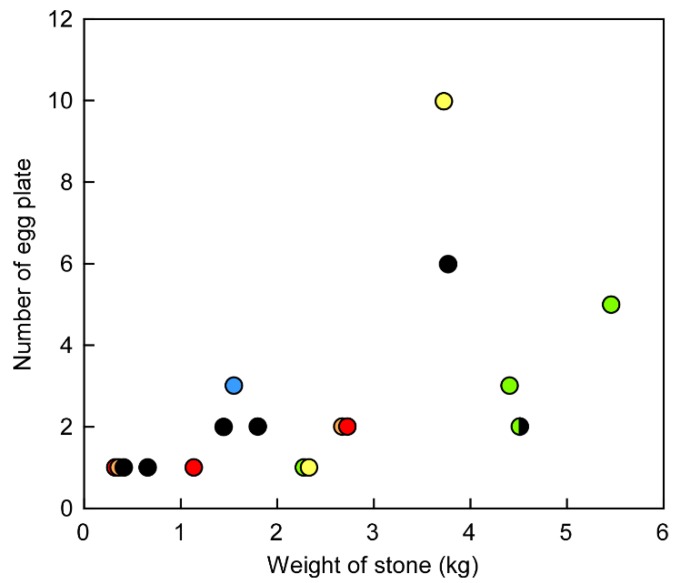
A plot showing the relationship between the weight of the rock and number of the flatworm *Planocera multitentaculata* egg plates deposited on it. Each color represents rocks collected from a different colony. Red, orange and yellow: 08 May 2019; green, blue and black: 22 May 2019.

Spawning colonies of the flatworm *P. multitentaculata* were found in a 100 m^2^ square region within the intertidal zone in Hayama, Miura Peninsula, Japan (Figure S1). Up to three adult flatworms and six rocks, with 1–10 egg plates per rock, were found within each spawning colony (Figure 1). The number of egg plates appeared to be a function of the rock size, with larger rocks harboring more egg plates. The smallest rock with an egg plate was 300–400 g and <100 mm in size (Figure 2).

We rearranged egg plate-bearing rocks that were obscured so that any egg plate feeding by pufferfish could be observed more easily. We found that several *T. alboplumbeus* specimens aggregated at the egg plate-bearing rocks during the incoming tide (Figure 1A). The receding tides revealed that the egg plates had been scraped off from the rocks where the pufferfish had aggregated (Figure 1C,D), suggesting that the pufferfish had fed on them.

**Figure 3:**
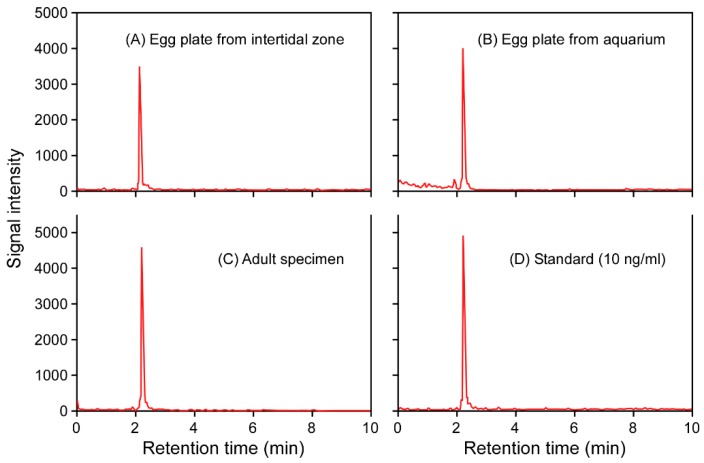
Typical mass chromatograms of the liquid chromatography with tandem mass spectrometry (LC-MS/MS) obtained under multiple reaction monitoring (MRM) mode (*m*/*z* 320 > 162). MRM patterns of extracts from the flatworm *Planocera multitentaculata* egg plates spawned in the wild (**A**), extracts from the flatworm egg plates spawned in the aquarium (**B**), extracts from the wild adult flatworms (**C**), and 50 ng/mL tetrodotoxin (TTX) standard (**D**).

Any remaining parts of the egg plates were collected and subjected to DNA sequencing and TTX extraction, followed by liquid chromatography with tandem mass spectrometry (LC-MS/MS) analysis. The 28S rRNA (LC489235–LC489236) and cytochrome *c* oxidase subunit I (COI) gene (LC489237–LC489238) fragment DNA sequences obtained were identical to those of the flatworm *P. multitentaculata* [32]. In addition, multiple reaction monitoring (MRM) patterns of the egg plate extracts were identical to that of standard TTX (Figure 3), and the toxin concentration was 23 ± 16 μg/g, which was highly significantly less than that in egg plates spawned in the laboratory (9393 ± 3356 μg/g) or in adult specimens (115 ± 52 μg/g) (*p* < 0.01; Table 1). The toxicity of egg plates from the wild was also apparently lower than the values reported in previous studies [30,31]. Since the TTX concentration of the flatworm larvae was equal or more than that of the flatworm egg plate, indicating that the toxin concentration of the egg capsule (jelly-like coat) itself was extremely low [31], our results show that the pufferfish ingested the flatworm embryos within the egg plates, targeting the toxic parts of the egg plates.

**Figure 4:**
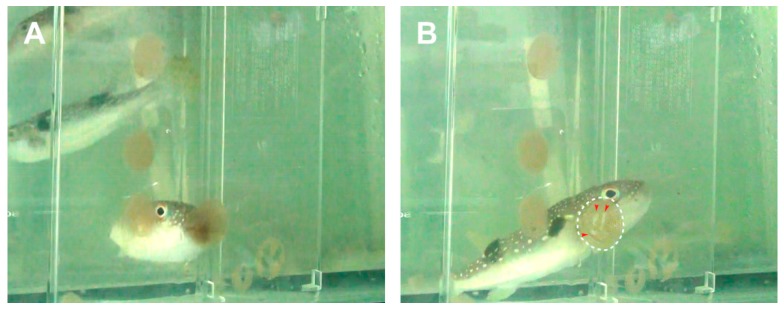
The pufferfish *Takifugu niphobles* (at present *Takifugu alboplumbeus*) feeding on the flatworm *Planocera multitentaculata* egg plates in the aquarium. Panel **A**, Multiple pufferfish feeding on egg plates on the surface of the plastic container. Panel **B**, Condition of the egg plate before and after being fed by the pufferfish. Red arrowheads indicate tooth marks made by the pufferfish. The video of the feeding behavior is provided as supplementary data (Video S2).

For the feeding experiment in the laboratory aquarium, adult *T. alboplumbeus* specimens were fed on the toxic planocerid egg plates deposited on the inside wall of transparent plastic containers (Figure 4). Highly concentrated TTX (9393 ± 3356 μg/g) was detected from the egg plate samples by LC-MS/MS analysis (Figure 3; Table 1), as in previous reports [30,31]. It is possible that the pufferfish use olfaction in detecting the toxic flatworm egg plates, since pufferfish have been reported to be able to smell TTX [33]. Interestingly, however, the pufferfish in our study tried to scrape the egg plates from the outer wall of the transparent plastic containers even though the egg plates had been deposited on the inner walls, suggesting that they (also) used visual cues to detect the egg plates. Further investigations are needed to understand this behavior. A control was introduced by allowing the pufferfish to feed on dried krill and artificial feed. The pufferfish ingest the toxic egg plates more positively than dried krill or artificial feed.

Previously, we had reported that *T. niphobles* (at present *Takifugu alboplumbeus*) juveniles and adults/young became toxic by feeding on *P. multitentaculata* larvae and adults, respectively [29]. Our findings in this study now show that the pufferfish also feed on *P. multitentaculata* egg plates, and thus, the flatworm egg plates appear to be an additional source of TTX. The flatworm egg plates deposited on rock and stone surfaces in the wild are known to contain highly concentrated TTX [30,31]. Our present study suggests that *P. multitentaculata* contributes to the toxification of *T. alboplumbeus* throughout its life, including the egg stage.

**Figure 5:**
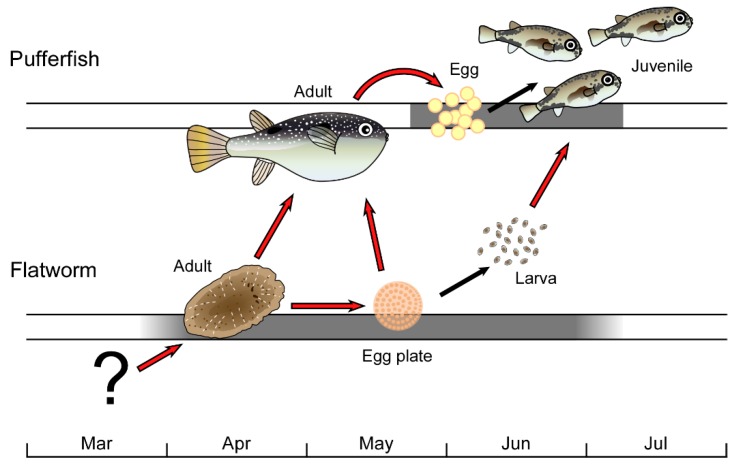
The life histories of the flatworm *Planocera multitentaculata* and the pufferfish *Takifugu niphobles* (at present *Takifugu alboplumbeus*), connected by the TTX present in the flatworm. The pufferfish ingests TTX by feeding on the flatworm egg plates (present study) and adults/young [29]. Red and black arrows indicate transfer of TTX and hatching, respectively. Horizontal gray bars represent the spawning season of the pufferfish (upper) and the toxic flatworm (lower).

Females of pufferfish in the genus *Takifugu* accumulate TTX in the ovaries, evidently to protect their larvae immediately upon hatching [34,35], although TTX might also be important to the pufferfish as a pheromone, as they appear to use it for aggregation during spawning [36,37]. As shown in Figure 5, *P. multitentaculata* spawn prior to *T. niphobles* (at present *Takifugu alboplumbeus*) in this region [31,37]. It is necessary that the pufferfish ingest TTX efficiently for the survival of the next generation [34,35], and the TTX source appears to be the flatworm and/or its egg plates.

*Planocera multitentaculata* deposit egg plates on unexposed sides of rocks in the intertidal zone [31,38]. These rocks varied in weight from a few hundred grams to a few kilograms. Although the rocks are generally too heavy for the pufferfish to overturn on their own, they are likely to be overturned by the force of the waves in the intertidal zone, and pufferfish risk entering the shallow waters before and during their spawning period to look for exposed *P. multitentaculata* egg plates. The start of the incoming tide might be the perfect opportunity for the pufferfish to feed on TTX-bearing organisms such as flatworms and ribbonworms or their eggs. On the other hand, the number of TTX-bearing organisms in this region is likely to be too low to account for the amount of TTX in the flatworm body [31]. Further studies are necessary to determine all the sources of TTX that endow the pufferfish with the potent toxicity that it is well known for [5,20].

## 3. Conclusions

In summary, the toxic planocerid flatworm *P. multitentaculata* spawns toxic egg plates on stones in the intertidal zone from spring to early summer. During this period, the toxic egg plates of the flatworm are fed upon by the pufferfish *T. alboplumbeus* (as confirmed by us in both the natural environment and glass aquaria). The flatworm spawns prior to the pufferfish in this region. These results suggest that the pufferfish utilize flatworm egg plates as an (additional) source of TTX with which to fortify themselves and the next generation.

## 4. Materials and Methods

### 4.1. Observation of Feeding Behavior in the Natural Environment

The feeding behavior of *T. niphobles* (at present *Takifugu alboplumbeus*) on *P. multitentaculata* egg plates deposited on rocks in the intertidal zone was observed at the start of the incoming tide (Hayama, Miura Peninsula, Japan; 35°15′N, 139°34′E; Figure S1). The stones with the flatworm egg plates were positioned such that the egg plates were easily visible. Observations were made on three separate occasions in April/May 2019, and recorded by video camera. The condition of the egg plates on the rocks before and after feeding were noted. Once the tide receded, the remaining egg plates were collected and stored at –30 °C for TTX and DNA extraction. The weight of the stones on which the flatworm deposited egg plates was measured by a handy electric balance in the field.

### 4.2. Feeding Experiment in the Laboratory

*Takifugu alboplumbeus* adults (*n* = 9, 79–106 mm standard length, 13.0–32.5 g body weight) were captured and reared for 2 weeks until the feeding experiment in the laboratory aquaria. *Planocera multitentaculata* adults (*n* = 6, 3.21–5.73 g body weight) were also captured and reared for 2 weeks until egg plates were deposited in plastic containers. The feeding experiment was carried out using wild *T. alboplumbeus* adults and toxic planocerid egg plates, dried krill or artificial feeds as food, in a 50-L glass aquarium with a circulating filtration system. The plastic containers with the flatworm egg plates deposited on them were placed in the aquarium containing the pufferfish, and the feeding behavior was observed and recorded by video camera. After feeding, the remaining egg plates were removed for TTX extraction.

### 4.3. PCR Amplification and DNA Sequencing

Total genomic DNA was extracted from egg plates following Tsunashima et al. [32]. DNA fragments corresponding to partial sequences of the 28S rRNA gene (approx. 1100 bp) and COI gene (approx. 600 bp) were amplified by PCR using pairs of primers HRNT-F2 (5′-AGTTC AAGAG TACGT GAAAC C-3′) / HRNT-R2 (5′-AACAC CTTTT GTGGT ATCTG ATGA-3′) and HRpra2 (5′-AATAA GTATC ATGTA RACTD ATRTC T-3′) / HRprb2-2 (5′-GDGGV TTTGG DAATT GAYTA ATACC TT-3′), respectively [32]. PCR amplification and sequencing of the PCR products were conducted following the protocol of our previous report [29]. The flatworm 28S rRNA and COI gene sequences have been submitted to the DDBJ/EMBL/GenBank databases under the accession numbers LC489235–LC489238.

### 4.4. LC-MS/MS Analysis

TTX extraction and LC-MS/MS analysis were done following the method used in a previous study [29]. The calibration curve was generated with 1–100 ng/mL of TTX standard (FUJIFILM Wako Pure Chemicals, Osaka, Osaka, Japan), which showed good linearity and precision (*y* = 28.7128*x* + 21.1116, *r*^2^ = 0.9911). The limit of detection value was calculated at 4.4 ng/mL for TTX.

### 4.5. Statistical Analysis

Data on TTX concentration from LC-MS/MS analysis were subjected to one-way analysis of variance (ANOVA) followed by the Tukey–Kramer post-hoc test, using StatView ver. 5.0 (SAS Institute Inc., Cary, NC, USA, 1999). A significance level was set at *p* < 0.01.

## Figures and Tables

**Table 1:** Toxicity of the flatworm *Planocera multitentaculata* at the adult and egg stages.

Sample	*n*	Body Weight (g)	Sample from:	TTX Concentration (μg/g)	TTX Amount (μg)
Adult	6	4.39 ± 0.92	Wild	115 ± 52 ^b^	523 ± 285
Egg plate	3	N/A	Wild	23 ± 16 ^a^	N/A
Egg plate	3	N/A	Aquarium	9328 ± 3356 ^a^	N/A

Remaining fragments of egg plates were collected after feeding by the pufferfish *Takifugu niphobles* (at present *Takifugu alboplumbeus*). Data are shown as mean ± standard deviation. Different superscripts (^a^ and ^b^) in TTX column indicate a significant difference among the measured values (Tukey–Kramer post-hoc test, *p* < 0.01).

## Supplementary Materials

The following are available online at https://www.mdpi.com/2072-6651/11/7/402/s1, Figure S1: Sampling site of the flatworm *Planocera multitentaculata* egg plates and observation of the pufferfish behavior. Arrow in the map indicates the specific location of the sampling site (the intertidal zone of Hayama, Miura Peninsula, Japan); Video S1: Predation process of the pufferfish *Takifugu niphobles* (at present *Takifugu alboplumbeus*) against the flatworm *Planocera multitentaculata* egg plates in the natural environment; Video S2: Predation process of the pufferfish *Takifugu niphobles* (at present *Takifugu alboplumbeus*) against the flatworm *Planocera multitentaculata* egg plates in the laboratory.
