# Supplementary material for: Toxic Flatworm Egg Plates Serve as a Possible Source of Tetrodotoxin for Pufferfish
Source: Toxins (Basel). 2019 Jul 11;11(7):402. doi: 10.3390/toxins11070402 (PMC6669758; doi:10.3390/toxins11070402)
Supplement: Supplementary file 1 [file toxins-11-00402-s001.zip › toxins-549022-SI.docx]

Supplementary Materials: Toxic Flatworm Egg Plates Serve as a Possible Source of Tetrodotoxin for Pufferfish

Taiki Okabe, Hikaru Oyama, Maho Kashitani, Yuta Ishimaru, Rei Suo, Haruo Sugita and Shiro Itoi


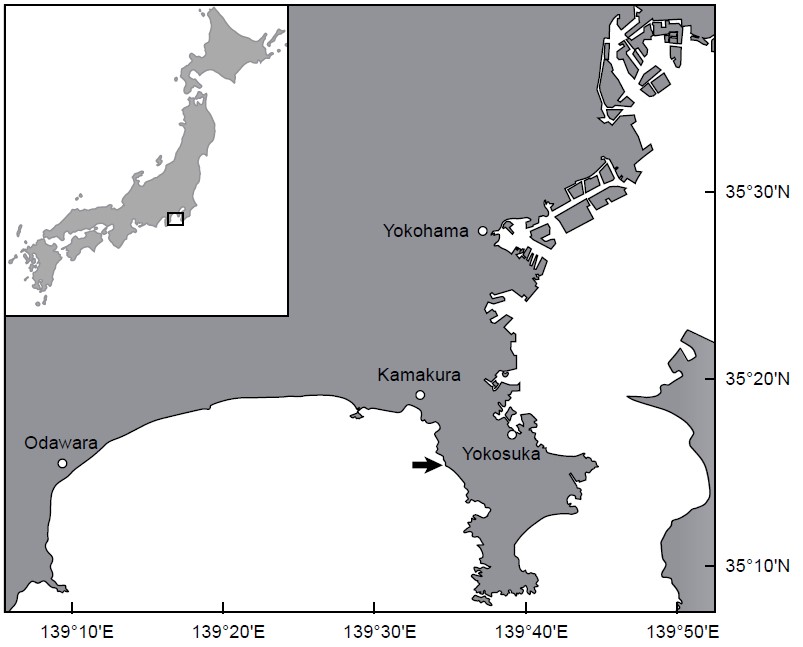


**Figure S1.** Sampling site of the flatworm *Planocera multitentaculata* egg plates and observation of the pufferfish behavior. Arrow in the map indicates the specific location of the sampling site (the intertidal zone of Hayama, Miura Peninsula, Japan).

**Video S1.** Predation process of the pufferfish *Takifugu niphobles* (at present *Takifugu alboplumbeus*) against the flatworm *Planocera multitentaculata* egg plates in the natural environment.

**Video S2.** Predation process of the pufferfish *Takifugu niphobles* (at present *Takifugu alboplumbeus*) against the flatworm *Planocera multitentaculata* egg plates in the laboratory.

.
