# Supplementary material for: Toxic Flatworm Egg Plates Serve as a Possible Source of Tetrodotoxin for Pufferfish
Source: Toxins (Basel). 2019 Jul 11;11(7):402. doi: 10.3390/toxins11070402 (PMC6669758; doi:10.3390/toxins11070402)
Supplement: Supplementary file 1 [file toxins-11-00402-s001.zip › toxins-549022-SI.pdf]

Supplementary Materials: Toxic Flatworm Egg Plates Serve as a Possible Source of Tetrodotoxin for Pufferfish

Taiki Okabe, Hikaru Oyama, Maho Kashitani, Yuta Ishimaru, Rei Suo, Haruo Sugita and Shiro Itoi

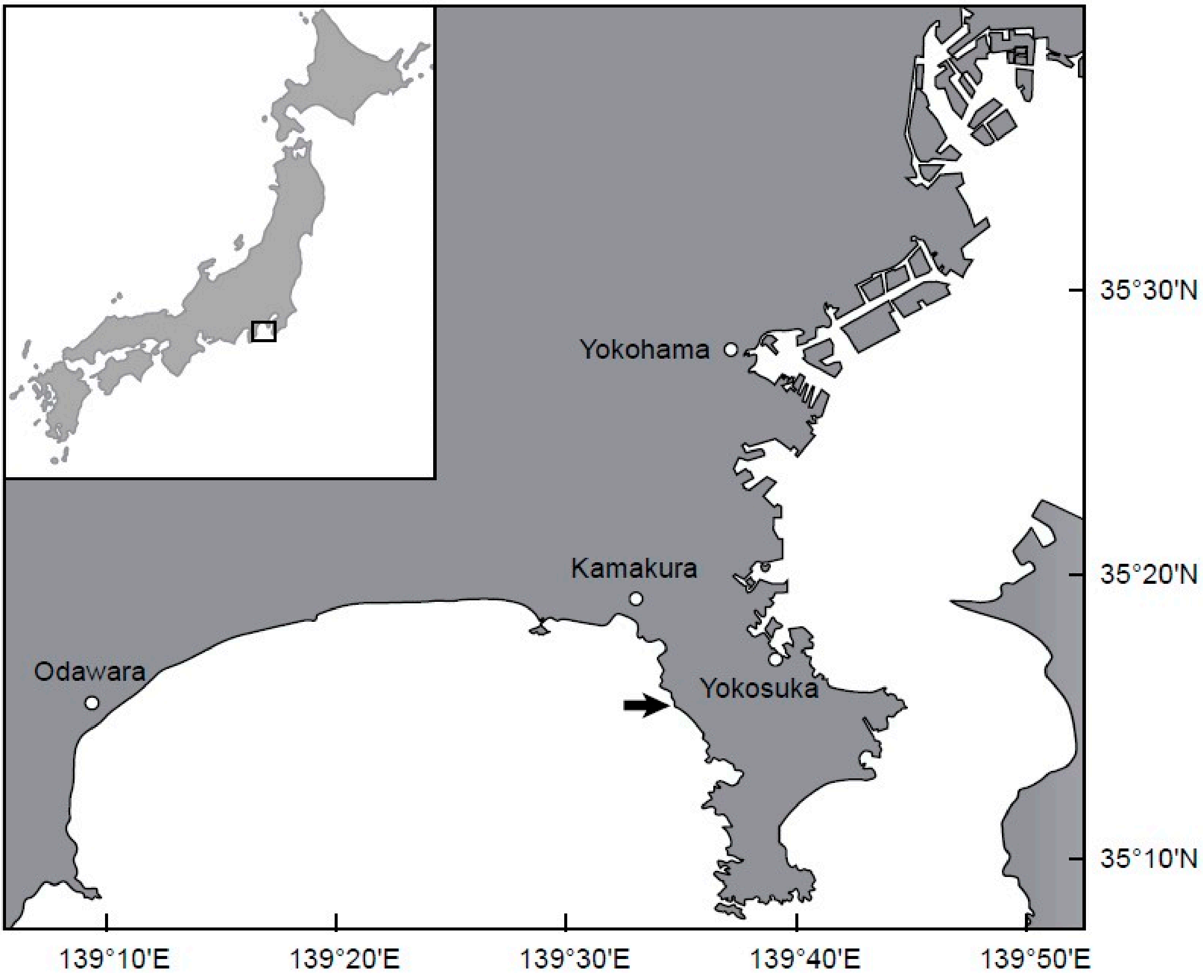

Figure S1. Sampling site of the flatworm *Planocera multitentaculata* egg plates and observation of the pufferfish behavior. Arrow in the map indicates the specific location of the sampling site (the intertidal zone of Hayama, Miura Peninsula, Japan).

Video S1. Predation process of the pufferfish *Takifugu niphobles* (at present *Takifugu alboplumbeus*) against the flatworm *Planocera multitentaculata* egg plates in the natural environment.

Video S2. Predation process of the pufferfish *Takifugu niphobles* (at present *Takifugu alboplumbeus*) against the flatworm *Planocera multitentaculata* egg plates in the laboratory.

.
